# WRKY transcription factors govern selenium metabolism in *Broussonetia papyrifera*: genome-wide identification, expression profiling, and regulatory network analysis

**DOI:** 10.1186/s12870-026-08407-y

**Published:** 2026-02-26

**Authors:** Huimin He, Qiangwen Chen, Shiming Deng, Jitao Li, Xin Cong, Shuiyuan Cheng, Weiwei Zhang, Feng Xu

**Affiliations:** 1 College of Horticulture and Gardening, Yangtze University, Jingzhou, Hubei 434025 China; 2 Hubei key Laboratory of Spices & Horticultural Plant Germplasm Innovation & Utilization, Yangtze University, Jingzhou, Hubei 434025 China; 3 Hubei Key Laboratory of Selenium Resource Research and Biological Application, Hubei Minzu University, Enshi, Hubei 445000 China; 4 Enshi Se-Run Material Engineering Technology Co., Ltd, Enshi, Hubei 445000 China; 5 National R&D Center for Se-rich Agricultural Products Processing, Wuhan Polytechnic University, Wuhan, Hubei 430023 China

**Keywords:** Selenium metabolism, qRT-PCR, Tissue specificity, WRKY, Subcellular localization

## Abstract

**Background:**

*Broussonetia papyrifera*, a novel forage tree species, exhibits a notable ability to accumulate selenium, making it a viable option for selenium enrichment feed. Nevertheless, the molecular mechanisms governing selenium metabolism in this tree are poorly understood, hindering its further development as a selenium accumulator.

**Results:**

This study revealed 47 *WRKY* transcription factors from the *B. papyrifera* genome. These WRKY transcription factors were characterized by their gene structure, protein domains, and synteny analysis across different species. Based on the phylogenetic analysis, these *WRKY* genes were categorized into three distinct subfamilies. By integrating transcriptomic and physiological data, nine *WRKY* genes were strongly correlated with selenium. Subsequently, quantitative qRT-PCR results further confirmed *BpWRKY34* and *BpWRKY25* as potential regulators of selenium metabolism in *B. papyrifera*, subcellular localization studies indicated that *BpWRKY34* and *BpWRKY25* were functional in the cell nucleus. Furthermore, we identified several miRNAs that potentially target *WRKY* family members. Co-expression network analysis revealed downstream enzymatic networks functionally linked to WRKY transcription factors in the selenium metabolic pathway of *B. papyrifera*.

**Conclusions:**

The integrated analysis of *BpWRKY* genes identified candidate genes that regulate selenium metabolism, offering a theoretical foundation for developing Se-enriched *B. papyrifera* through genetic improvement.

**Supplementary Information:**

The online version contains supplementary material available at 10.1186/s12870-026-08407-y.

## Background

In response to the escalating demand for livestock and poultry products, traditional feed supplies are inadequate for meeting the developmental requirements of the livestock industry. Consequently, there is an immediate need to introduce innovative feed sources to address this challenge [1]. Some studies suggest that unconventional feeds can serve as animal feed without negatively affecting development. However, the limited research on such feeds’ nutritional value and utility hinder their widespread application in the livestock industry [2]. *B. papyrifera* is a deciduous tree of the Moraceae family, which has the characteristics of wide adaptability, strong stress resistance, strong disease resistance and rapid growth. Its leaves are abundant in crude protein and fat, surpassing traditional forage feeds such as alfalfa and mulberry in nutrient content [3], thus demonstrating its potential as a novel animal feed. Research indicates that incorporating 15% *B. papyrifera* silage into cattle feed enhances the final weight of beef cattle, improves the nutritional content of meat, and increases polyunsaturated fatty acids (PUFA) and docosahexaenoic acid (DHA) levels [4]. Furthermore, fermented *B. papyrifera* feed can enhance chicken feed intake, egg yolk color, and lipid metabolism, thereby improving egg quality [5].

Selenium is an essential trace element for human health that plays a critical role in maintaining various tissues and organs, including the heart [6], blood [7], and bones [8], and in adjunctive cancer treatment [9–12]. Selenium deficiency can have severe health consequences, leading to diseases such as Keshan disease [13, 14], diabetes [15, 16], and Kashin-Beck disease [17]. Under selenium-depleted conditions, the brain prioritizes its own needs, leading to irreversible damage to other organs [18]. Due to geographical variations, the global selenium distribution is uneven [19], with humans primarily obtaining selenium through their diet and livestock and poultry products being significant sources.

Previous studies have revealed that *B. papyrifera* has a strong capacity for selenium enrichment [20]. Through exogenous selenium fortification, not only does the total selenium content in *B. papyrifera* significantly increase, but the contents of soluble sugars, phenolic acids, and flavonoids increase, as does the increase in antioxidant enzyme activity [20]. While comparative transcriptomics studies have identified genes related to selenium synthesis involved in selenium metabolism in *B. papyrifera*, the regulatory mechanisms of selenium metabolism in this species have largely not been identified.

Studies indicate that transcription factors play pivotal roles in plant selenium metabolism. In rice, Se influences the expression of *MYB* and *WRKY* transcription factors in response to arsenic stress [21], and in tea plants, the expression of the *MYB* and *bZIP* transcription factors are upregulated under selenite treatment [22]. Additionally, ethylene response factors (ERFs) have been demonstrated to respond to selenate treatment, and *Arabidopsis* plants overexpressing *ERF1* have been observed to exhibit increased tolerance to selenate [23]. In *Arabidopsis*, *AtWRKY47* may regulate the expression of *HMT1* and *HMT3* and thus selenium sensitivity in plants by interacting with one or more factors [24, 25]. Previous studies have demonstrated that selenate treatment significantly modulates the accumulation of secondary metabolites in *Cardamine violifolia* [26], while concurrently inducing a marked upregulation of sulfate transporter (SULTR) genes and sulfur assimilatory enzyme-related genes [27]. Moreover, a recent study found that the sequences of the *CvWRKY021* and *CvWRKY099* genes in *Cardamine violifolia* are highly similar to the *AtWRKY47* sequence and are defined as key regulators of selenium enrichment in *C. violifolia* [28]. MicroRNAs (miRNAs) are typically located at the post-transcriptional level, where they function in the process of gene silencing. In *Helianthus annuus*, miR396 targets and silences *HaWRKY6* expression [29]. In apple, Md-miR156ab and Md-miR395 repressed the expression of the transcription factor genes *MdWRKYN1* and *MdWRKY26* and reduced the expression of several pathogenesis-related (PR) genes, resulting in susceptibility of the susceptible apple variety GD to ALT1 [30].

In this study, all members of WRKY family were identified from the genome of *B. papyrifera* and studied in terms of distribution in chromosomes, chromosomal collinearity, conserved motifs, structural domains, and phylogenetic analysis. The correlation between *WRKY* gene expression and the selenium content was analyzed based on the RNA-seq results, identifying potential candidate *BpWRKYs* involved in regulating the selenium metabolism of *B. papyrifera*. The tissue expression patterns of these genes were also analyzed. The miRNAs targeting WRKY were also identified and the regulatory pattern of the targeted pairs was verified by Quantitative Real-time polymerase chain reaction (qRT-PCR) experiments. In addition, subcellular localization assays were performed for the candidate BpWRKY. Our study aimed to identify and screen candidate *BpWRKY* transcription factors involved in selenium metabolism in selenium enrichment *B. papyrifera*, to provide genetic resources and a solid theoretical basis for selenium uptake and transformation in selenium enrichment trees, and to provide new insights for genetic improvement of selenium enrichment *B. papyrifera* varieties.

## Results

### Identification of *WRKY* gene families in *B. papyrifera*

Forty-seven sequences containing the conserved *WRKY* domain were discerned within the *B. papyrifera* genome. Examination of their physicochemical properties revealed that the amino acid count of the *BpWRKYs* ranged from 183 to 752 (Additional file 1: Table S1). The relative molecular weights varied between 21.33 and 81.80 kDa and generally correlated with the amino acid content. The isoelectric points ranged from 4.90 to 9.83, with 18 proteins exhibiting basic characteristics (pI > 7) and 28 displaying acidic properties (pI < 7). Except for *BpWRKY4*, which was identified as an unstable protein, the remaining proteins exhibited instability indices below 40, indicating their stability. Hydrophilicity analysis indicated that all 47 *BpWRKYs* are hydrophilic proteins. Subcellular localization predictions indicated that all the *BpWRKY* proteins were localized within the cell nucleus.

### Phylogenetic tree of the *WRKY* family in different plant species

**Fig. 1:**
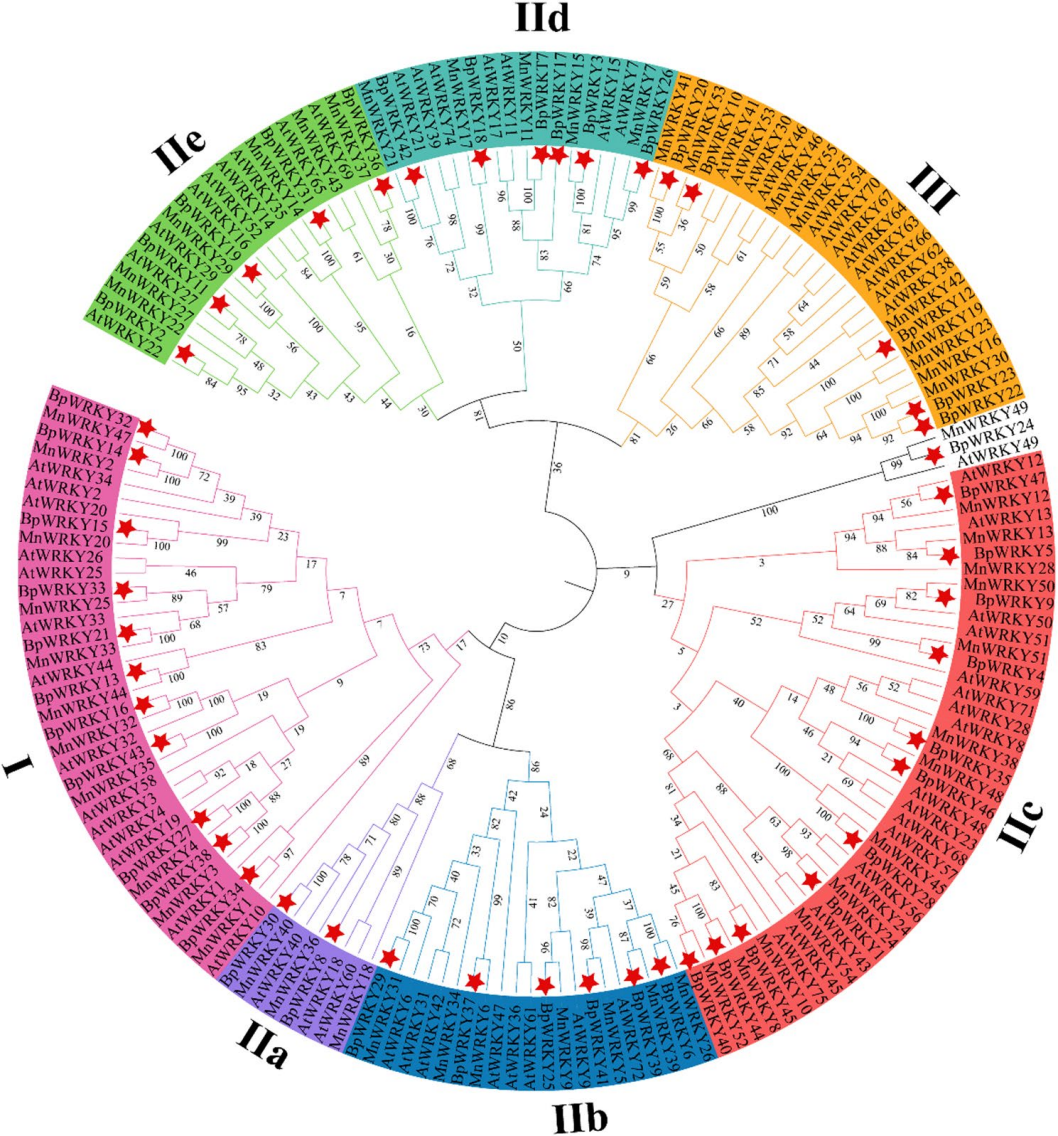
Phylogenetic tree of WRKY families in *B. papyrifera*, *M. alba*, and *A.thaliana*. BpWRKYs were marked by red stars

A phylogenetic tree was constructed utilizing WRKY transcription factors from *(A) thaliana*, *M. alba*, and *(B) papyrifera* (Additional file 2: Table S2). Consistent with previous reports [31], all identified *B. papyrifera* WRKY contained the conserved WRKYGQK domain. Based on their WRKY domain architecture [32] and zinc finger motifs [33], these transcription factors were classified into three major groups (I, II, and III; Fig. 1).

Group I consisted of 10 members characterized by two complete WRKY domains (N- and C-terminal) and C2H2-type zinc finger structures. The 33 Group II members were further divided into five subgroups (IIa-e) according to their clustering patterns. Subgroups IIa, IId, and IIe shared a C-X5-C23-HXH zinc finger motif, while IIb and IIc exhibited distinct patterns (C-X6-C29-HXH and C-X4-C23-HXH, respectively). Group III contained three members featuring a single C2HC-type zinc finger. Notably, BpWRKY24 showed divergent characteristics and could not be assigned to any established group. This classification scheme is largely consistent with that reported for the *WRKY* family in *Morus alba* [34].

### Conserved motif, gene structure and distribution, and domain analysis

**Fig. 2:**
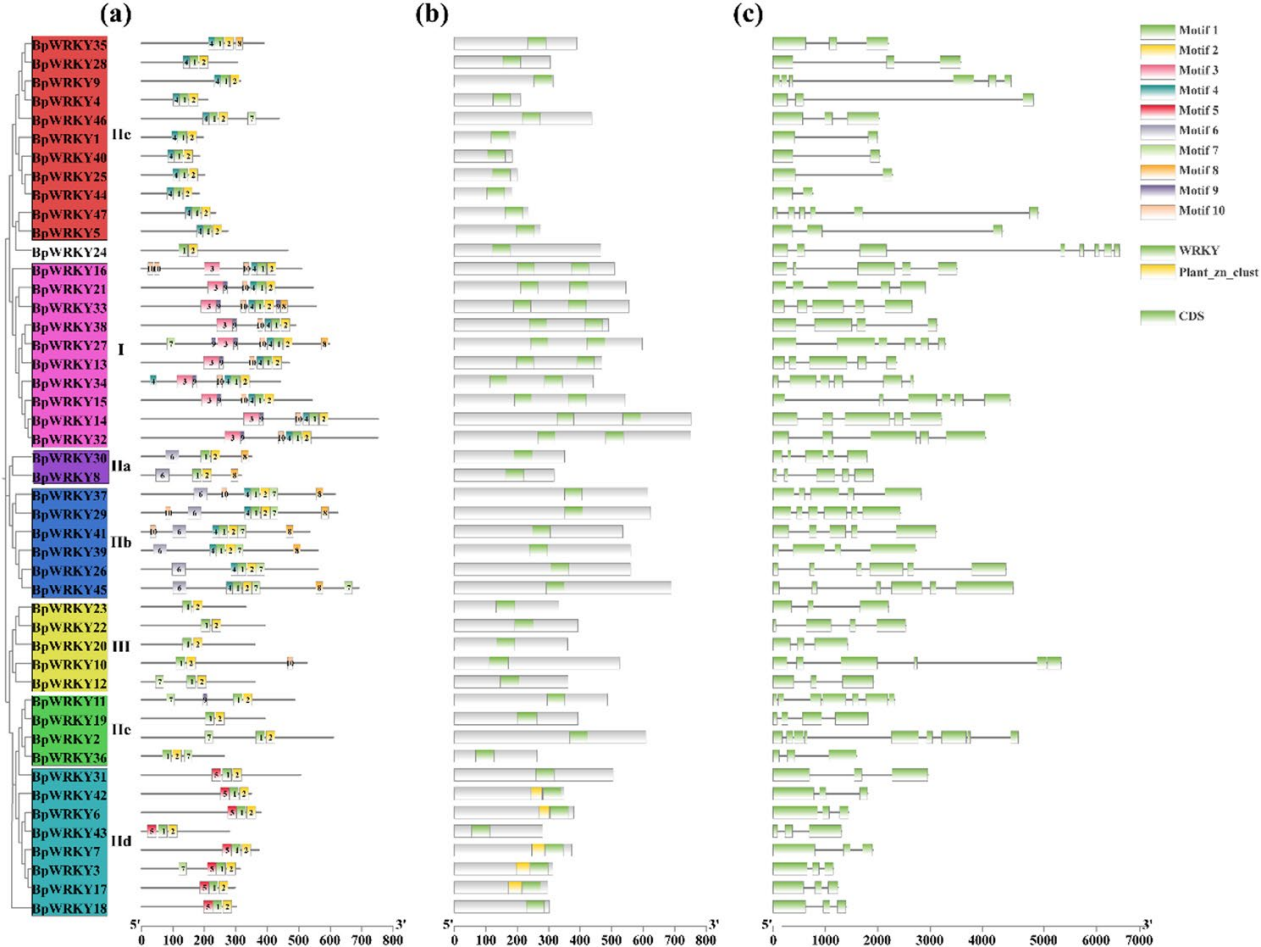
Conserved motif distribution, conserved domain, and gene structure of WRKY families in *B. papyrifera*. (**a**) is conservative motif; (**b**) is protein conserved domain; (**c**) is gene structure

The MEME tool was used to analyze conserved motifs within the *B. papyrifera WRKY* transcription factor family, resulting in the identification of ten motifs exhibiting high levels of conservation (Fig. 2a). The significance of a motif in the WRKY protein sequence is primarily determined by its frequency of occurrence within the sequence. Among these motifs, motif 1 corresponds to the *WRKY* motif [35] and is notably conserved across the entire *BpWRKY* family. Motif 2 corresponds to the zinc finger structure, and notably, all the *BpWRKY* transcription factors included both Motif 1 and Motif 2. Additionally, Motif 3 represents the conserved domain of *WRKY* within evolutionary group I, while Motif 4 is present in the protein structures of genes belonging to evolutionary groups I, IIb, and IIc.

Analysis of conserved domains revealed that every WRKY protein harbors the signature WRKY domain, and members of the IId subfamily additionally possess a Plant-Zn-clust domain (Fig. 2b). Examination of gene structures showed that all *BpWRKY* genes contain multiple introns (Fig. 2c), with exon counts predominantly falling into two categories—three or five exons. More precisely, group I genes carry 5–6 exons. the broader group II spans 2–9 exons. subgroups IIa and IIb each have 5–6 exons; subgroups IIc and IId uniformly exhibit three exons; subgroup IIe ranges from 3 to 9 exons; and group III members contain 3–6 exons.

### Chromosomal localization and collinearity analysis of *BpWRKY* genes

**Fig. 3:**
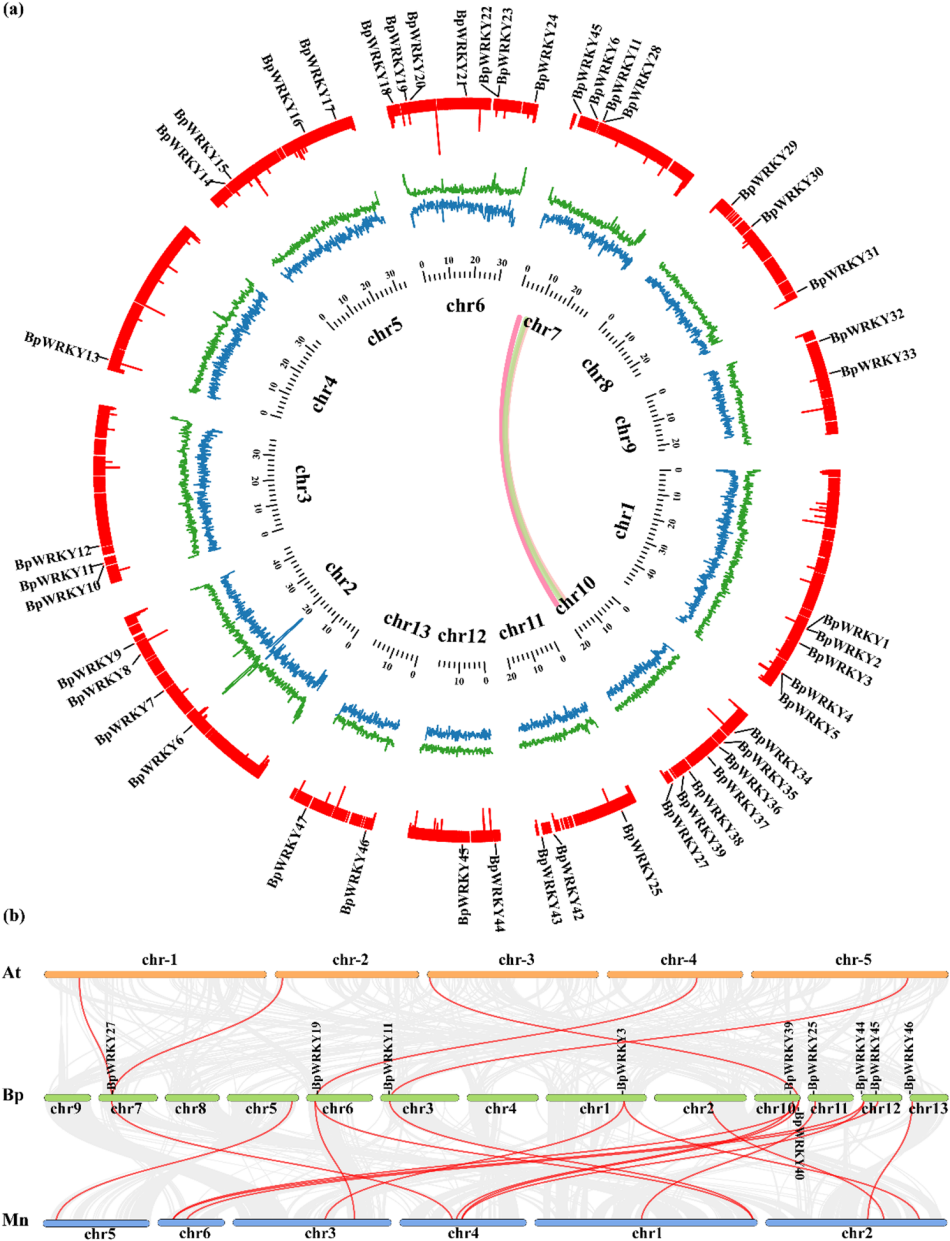
Comparative collinearity analysis of *WRKY genes* in *B. papyrifera* and other species. (**a**) The interchromosomal relationships of the *B. papyrifera WRKY* genes are depicted in a circular map, illustrating chromosome density, GC content, and base position. The outermost circle represents the localization of *WRKY* genes on chromosomes. Circle 1 is blue and indicates chromosome density, circle 2 is green and represents GC content, while circle 3 is red and signifies the distribution of unknown bases on the chromosome. (**b**) Collinearity analysis of *WRKY* genes between *B. papyrifera* (Bp), *A.thaliana* (At), and *M. alba* (Mn)

Chromosomal localization analysis revealed that the 47 members of the *BpWRKY* family were dispersed randomly across 13 chromosomes (Fig. 3a). Among these, chromosomes 6 and 10 each accommodate seven genes, while chromosome 4 houses the fewest, with only one gene. Notably, a higher GC content implies greater DNA stability. Chromosome 2 corresponds to the area of greatest gene density. According to the inner synteny analysis of the *B. papyrifera* genome, three pairs of *BpWRKYs* exhibiting high duplication levels were identified, indicating their involvement in significant segment duplications within the genome.

Interspecies synteny analysis revealed the presence of five pairs of homologous genes between *(A) thaliana* and *(B) papyrifera*, among which one pair included an Arabidopsis gene *AT1G13960*, *AT2G03340* that was homologous to *BpWRKY27*. In the case of *M. alba* and *B. papyrifera*, 16 pairs of homologous genes were identified, with multiple *M. alba* genes identified as homologs to a single *B. papyrifera* gene (*BpWRKY3*, *BpWRKY19*, *BpWRKY40*, *BpWRKY44*; Fig. 3b, Additional file 3: Table S3). These findings suggest that this gene family’s expansion may have occurred before the divergence of *B. papyrifera*.

### Identification of cis-acting elements in *BpWRKY* promoter regions

**Fig. 4:**
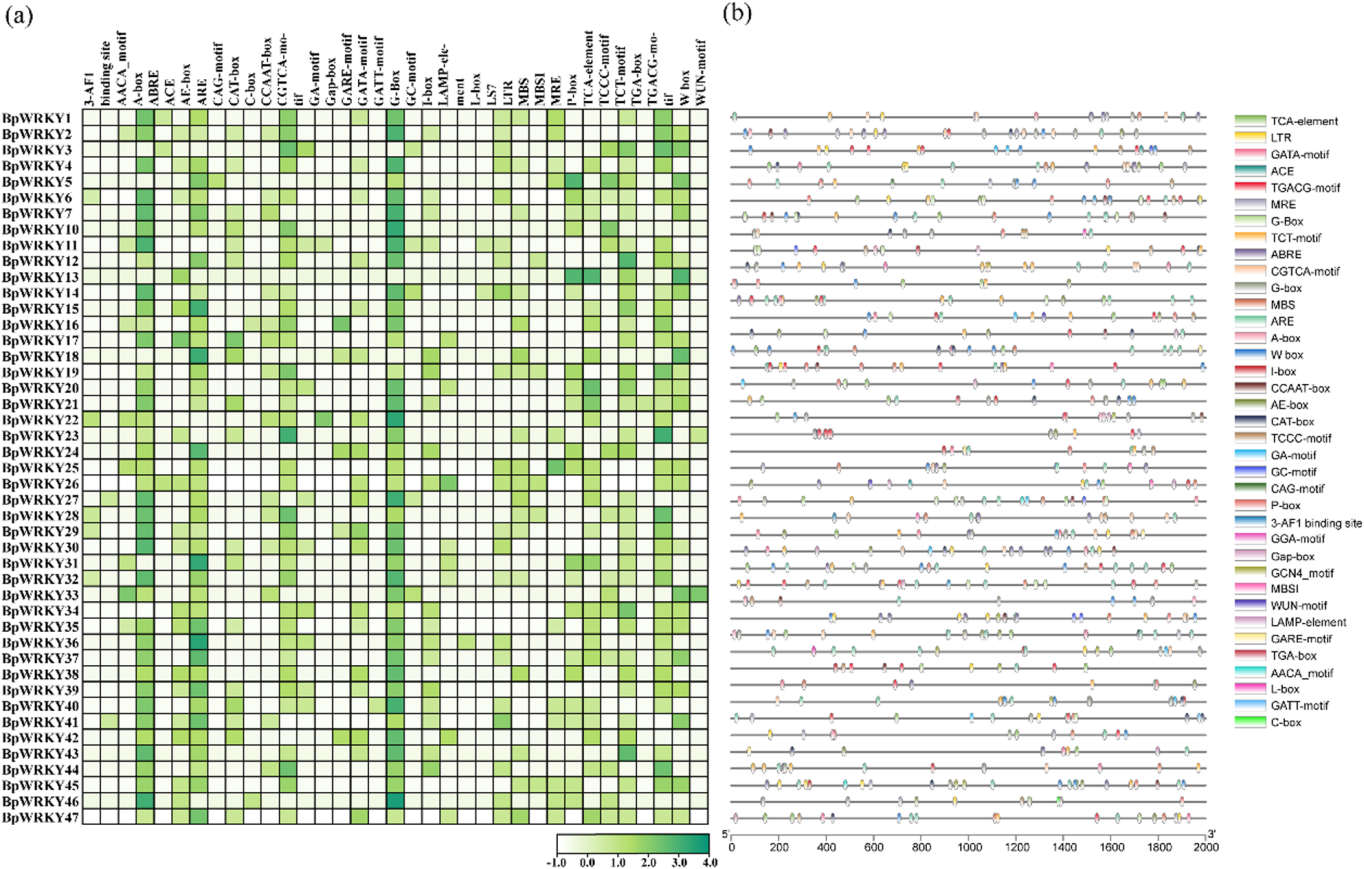
Analysis of *cis-*acting elements of *BpWRKY* promoters. (**a**) *BpWRKYs cis-*element heat map; (**b**) the original site visualization

Comprehensive analysis of the 2-kb promoter regions upstream of *BpWRKY* genes revealed 7,235 cis-acting elements representing 105 distinct types (Fig. 4a). Following the removal of uncharacterized elements, the remaining functional cis-regulatory elements were systematically categorized and visualized through heatmap analysis.

The promoter regions of all 47 *BpWRKY* genes contained multiple functionally important cis-elements, which were classified into three major categories: (1) Light-responsive elements, including G-box and MYB-binding sites (MRE) involved in light signaling; (2) Hormone-responsive elements, encompassing abscisic acid-responsive (ABRE), methyl jasmonate-responsive (CGTCA-motif), salicylic acid-responsive (TCA-element), and auxin-responsive (AuxRR-core) elements; (3) Stress-responsive elements, featuring low-temperature responsive (LTR), anaerobic induction (ARE), and drought-inducible MYB-binding sites (MBS). Notably, the co-occurrence of MRE and MBS elements suggests potential functional interactions between *BpWRKY* transcription factors and *MYB* proteins, which may form regulatory complexes to modulate gene expression under various physiological conditions.

### Differential regulation of *BpWRKY* genes in response to selenium treatment

**Fig. 5:**
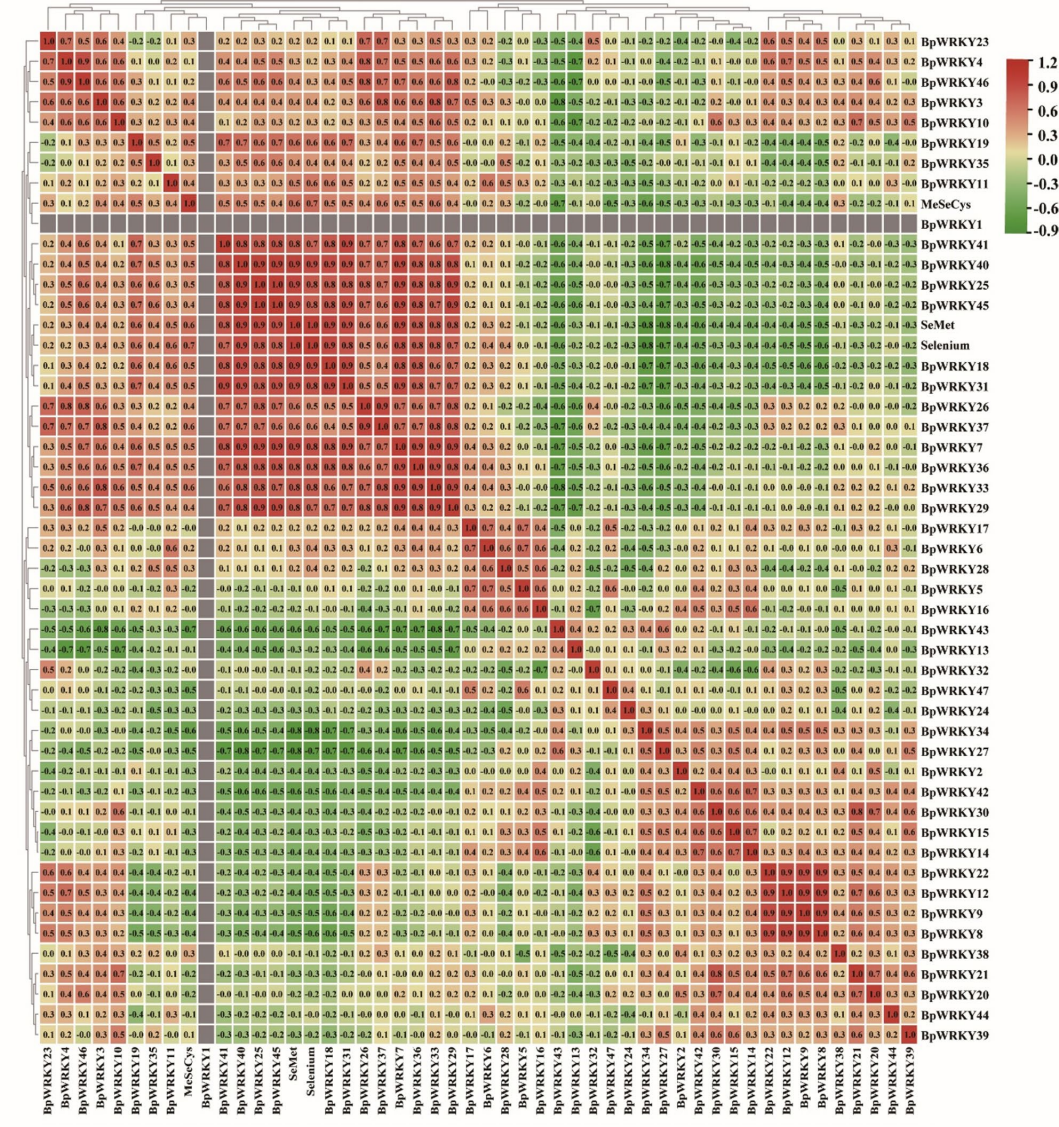
Heatmap of the FPKM value matrix of *BpWRKYs*

**Fig. 6:**
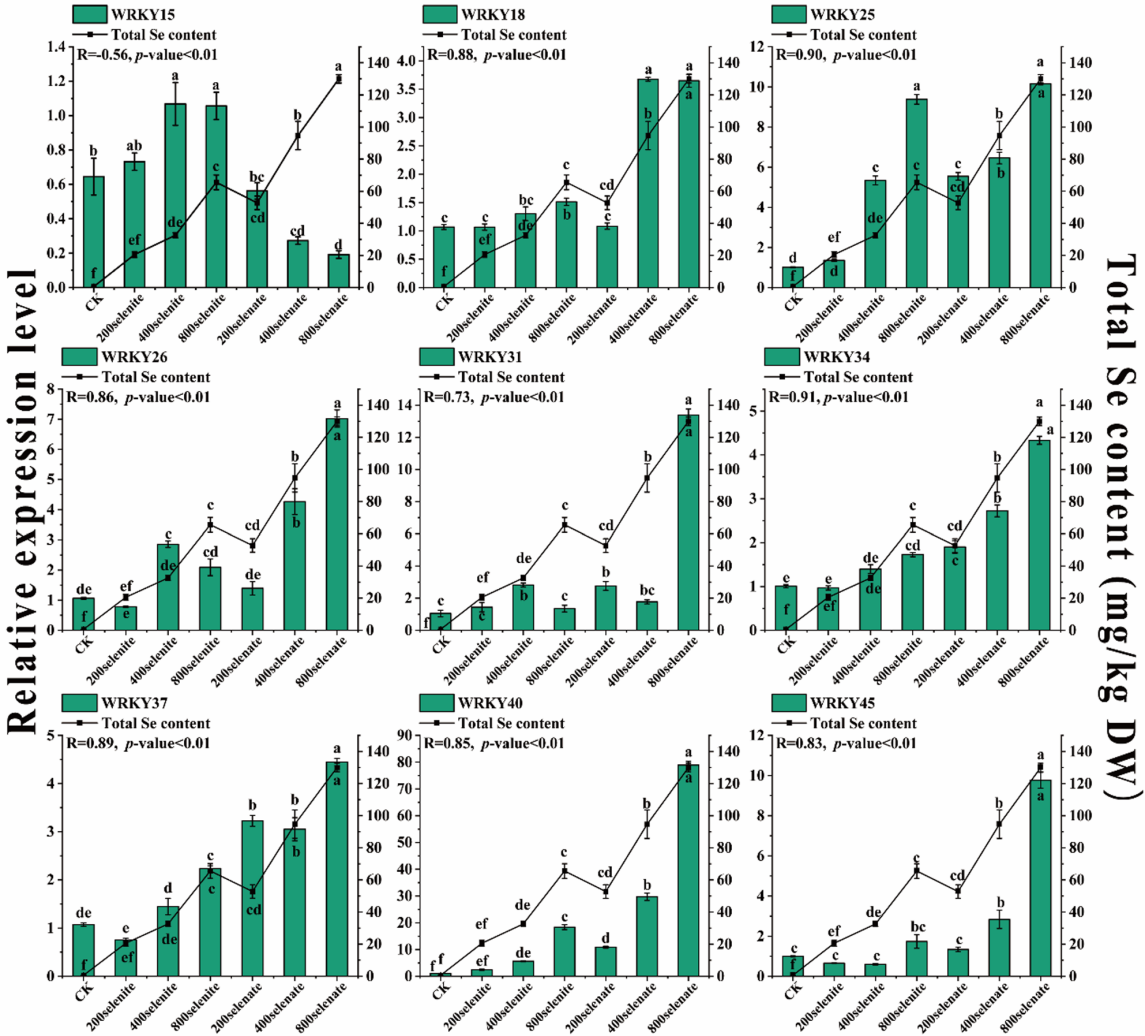
The expression patterns of 9 *BpWRKYs* under different forms of selenium were examined. The correlation between the expression levels of *BpWRKYs* and selenium content was analyzed. The horizontal axis represents the various treatments, while the left Y-axis indicates the relative expression levels of *BpWRKYs* and the right Y-axis represents the total selenium content

Based on transcriptomic analysis, we identified nine selenium-responsive genes showing strong correlation with selenium metabolism (Fig. 5). These candidate genes were subsequently analyzed by qRT-PCR to examine their expression patterns under different concentrations of selenate and selenite treatments. (Additional file 4: Table S4, Additional file 5: Table S5). Quantitative analysis revealed significant positive correlations (*r* = 0.56–0.91) between *BpWRKY* gene expression and selenium enrichment (Fig. 6). Among these, *BpWRKY34* (*r* = 0.91, *P* < 0.05) and *BpWRKY25* (*r* = 0.90, *P* < 0.05) demonstrated the strongest correlations, showing dose-dependent upregulation with increasing selenite concentrations. In contrast, *BpWRKY26* and *BpWRKY45* exhibited preferential induction by selenate treatment. Notably, *BpWRKY26* displayed a biphasic response to selenite, with expression peaking at 400 µM before declining, suggesting potential feedback regulation. Similarly, *BpWRKY31* showed transient induction under selenite treatment, though without subsequent downregulation, indicating its positive but potentially saturable involvement in selenium response.

The majority of examined genes exhibited consistent positive regulation under selenium treatment. However, *BpWRKY15* presented an exceptional case, showing contrasting responses to different selenium forms a positive correlation with selenate but a negative correlation with selenite, implying potential speciation-dependent regulatory mechanisms.

### Tissue-specific expression pattern of *BpWRKYs* under selenium stress

**Fig. 7:**
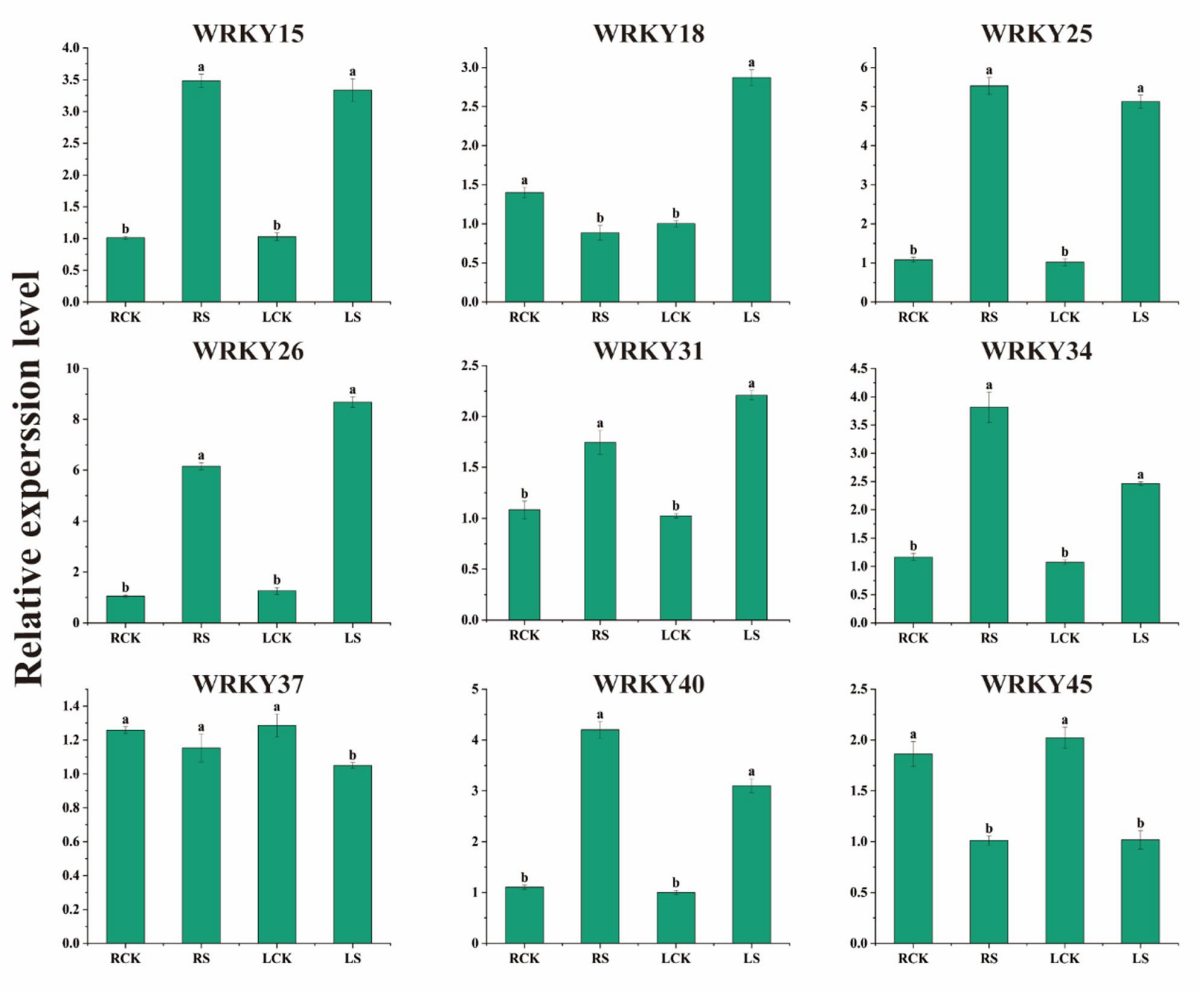
Tissue-specific expression pattern of 9 *BpWRKYs* under sodium selenate treatment. RCK was the root sample of the control, RS was the root sample treated with 400 mg/L selenium, LCK was the leaf sample of the control, LS was the leaf sample treated with 400 mg/L selenium, and the columns labeled with different letters had significant differences (*p* < 0.05), data were calculated using 2^-ΔΔCt^ method

Expression analysis of nine *WRKY* genes in roots and leaves revealed three distinct response patterns to selenium stress (Fig. 7). First, compared with those in the controls, the expression of six WRKYs (*BpWRKY15*, *BpWRKY25*, *BpWRKY26*, *BpWRKY31*, *BpWRKY34*, and *BpWRKY40*) in both the roots and leaves of plants under 400 mg/L selenate stress increased, indicating that these transcription factors play positive regulatory roles in the selenium stress response in both tissues. Second, differential expression patterns were observed, as exemplified by the expression of *BpWRKY18*, which exhibited a significant decrease in expression in roots but a nearly threefold increase in expression in leaves, suggesting opposing roles in these tissues. Third, genes such as *BpWRKY37* and *BpWRKY45* exhibited downregulated expression in both the roots and leaves, with *BpWRKY45* exhibiting significant downregulation in both, suggesting a negative regulatory role in the selenium tolerance of *B. papyrifera*.

### Screening of miRNA that targets *BpWRKYs*

**Fig. 8:**
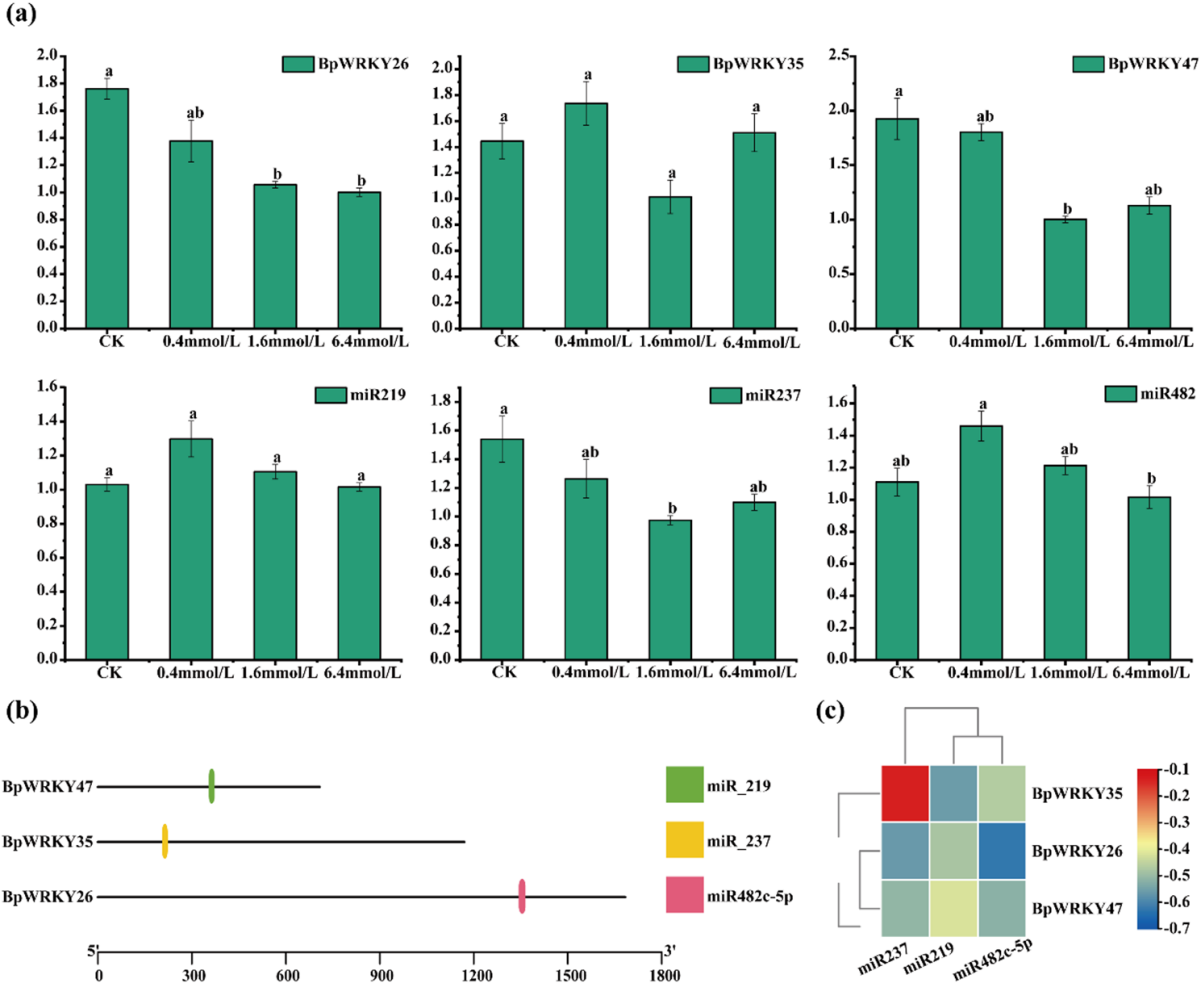
(**a**) Histograms of *WRKYs* vs. miRNA expression, (**b**) map of predicted cleavage sites of miRNAs that target *WRKYs*, (**c**) correlation matrix of miRNAs that target *WRKYs*

To elucidate the post-transcriptional regulation of *WRKY* transcription factors, we performed computational prediction of miRNA target sites using the psRNATarget platform (https://www.zhaolab.org/psRNATarget/home). Bioinformatic analysis revealed three potential miRNA-WRKY regulatory pairs: miR482c-5p::BpWRKY26, miR_237::BpWRKY35, and miR_219::BpWRKY47 (Fig. 8b). qRT-PCR validation demonstrated inverse expression patterns between these miRNAs and their corresponding target genes (Fig. 8a, c), with miR482c-5p exhibiting the strongest negative correlation with *BpWRKY26* expression. Notably, the miR482c-5p::BpWRKY26 pair was of particular interest given BpWRKY26’s established role in selenium metabolism. This inverse relationship suggests that miR482c-5p may modulate *B. papyrifera*’s selenium stress response through targeted suppression of *BpWRKY26* expression.

### Analysis of selenium-related downstream target gene regulation

**Fig. 9:**
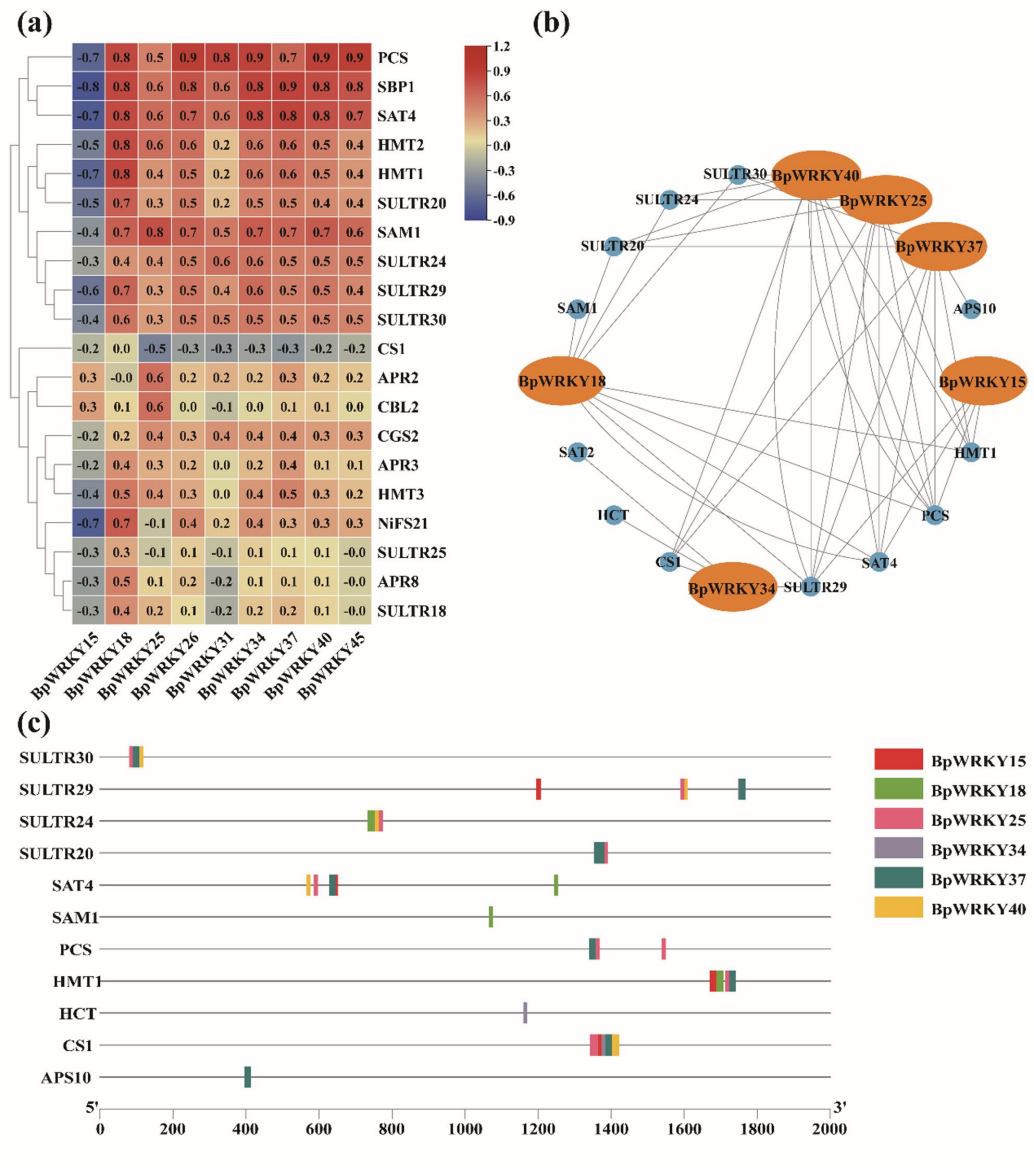
(**a**) Fluorescence quantitative heat map and interaction network of *BpWRKYs* with functional genes. Red represents positive correlation, blue borders negative correlation, the darker the color the higher the correlation with numerical values. (**b**) Transcription factor (TF) related networks, orange is *BpWRKY*, blue is downstream structural genes, and the lines indicate single regulatory predictions from orange to blue. (**c**) The line pattern indicates the promoter length, and the position of the gene colour block on the line indicates the position of the promoter that regulates the gene

qRT-PCR analysis revealed strong correlations (|r| > 0.6) between candidate selenium-associated *BpWRKY* genes and key selenium metabolic enzymes (*PCS*,* SBP1*,* SAT4*), with moderate correlations (|r| ≥ 0.5) observed for HMT2, HMT1, and SULTR20 (Fig. 9a). Co-expression network analysis revealed an extensive regulatory relationship between *BpWRKY* transcription factors and multiple selenium metabolism-related genes, including sulfur transporters (SULTR20, SULTR24, SULTR29, SULTR30), Selenocompound synthesis enzymes (APS10, HMT1), and cysteine metabolism genes *(PCS*, *SAT2*, *SAT4*, *CS1*, *HCT*, *SAM1*) (Fig. 9b).

Notably, five *BpWRKY* genes (*BpWRKY15*,* 18*,* 34*,* 37*,* 40*) emerged as central regulators in the current network. Among these, *BpWRKY40* displayed the broadest regulatory scope, potentially controlling up to 10 downstream targets, while *BpWRKY34* regulated at least 4 target genes. Promoter analysis demonstrated that most *BpWRKYs* share common binding sites on their target genes (Fig. 9c), suggesting coordinated regulatory mechanisms. For example, *SULTR30* is co-regulated by *BpWRKY25*, *37*, and 40; *HMT1* by *BpWRKY15*, *18*, *25*, and *37*; and *CS1* by a collaborative module of *BpWRKY15*, *25*, *34*, *37*, and *40*.

### Subcellular localization of *BpWRKY34* and *BpWRKY25*

The gene expression of *BpWRKY34* and *BpWRKY25* exhibited a significant correlation with selenium content, with correlation coefficients of 0.91 and 0.90, respectively. It is indicated that these two *WRKY* genes are likely to be involved in selenium metabolism regulation in *B. papyrifera* and respond to selenium regulation in *B. papyrifera*. Consequently, *BpWRKY34* and *BpWRKY25* were selected as candidate genes for the study of selenium expression in *B. papyrifera*.

**Fig. 10:**
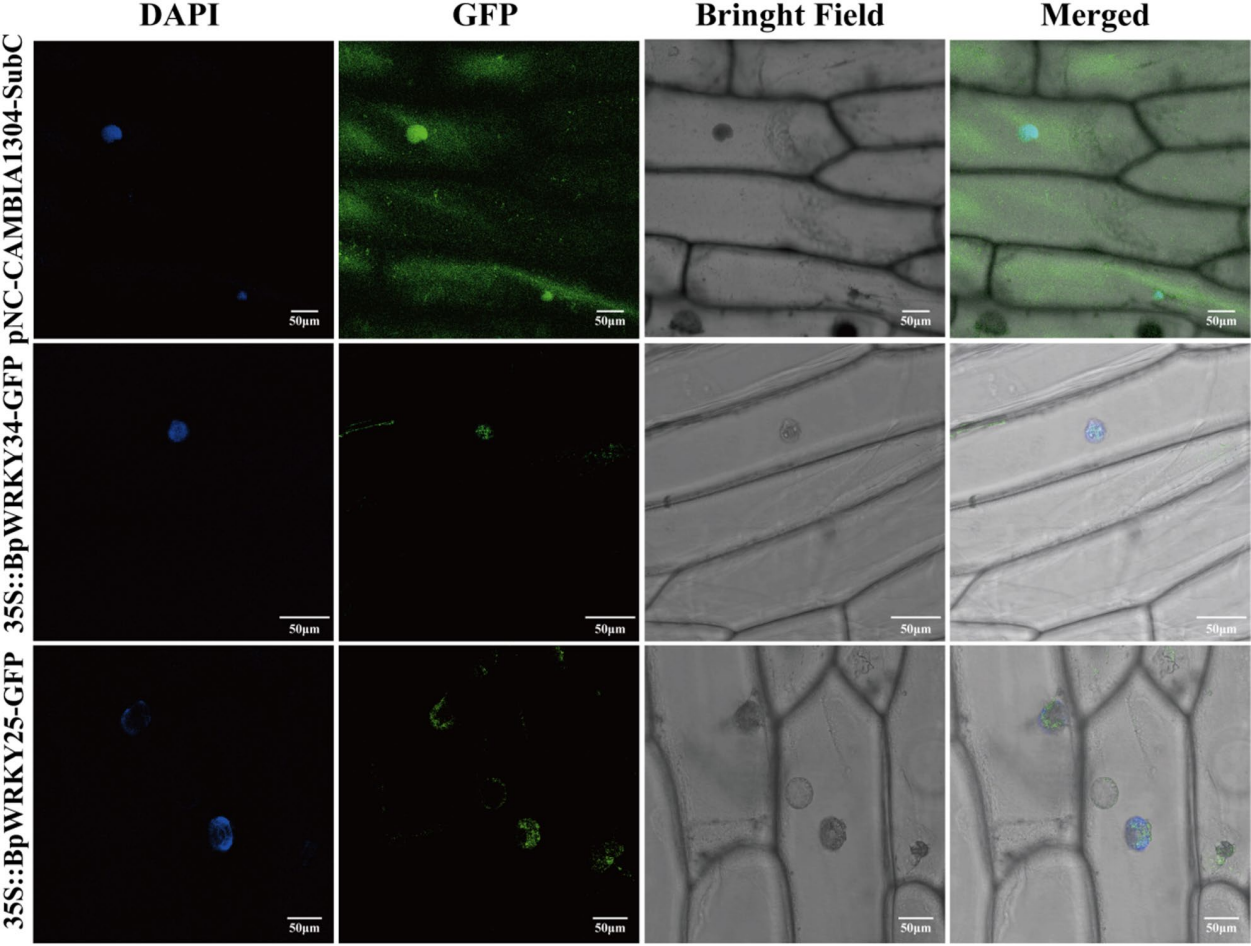
Subcellular localization of *BpWRKY34* and *BpWRKY25* in *A. cepa*. pNC-CAMBIA1304-SubC is the positive control, 35 S::BpWRKY34-GFP is the fusion protein of BpWRKY34 and GFP report protein, 35 S::BpWRKY25-GFP is the fusion protein of *BpWRKY25* and GFP report protein. DAPI staining agent for blue fluorescence as a control of the location of the nucleus

Regarding the subcellular localization of *BpWRKY34* and *BpWRKY25*, fusion expression vectors for the two genes displaying the highest correlation with selenium expression were constructed for subcellular localization experiments in onion epidermal cells. The results revealed that the green fluorescent signal of the positive control was detectable in both the nucleus and the cytoplasm (Fig. 10). In contrast, the fluorescence signals of the BpWRKY34-GFP and BpWRKY25-GFP fusion proteins were exclusively detected in the nucleus, revealing the nuclear localization of the *BpWRKY34* and *BpWRKY25* proteins.

## Discussion

*B. papyrifera* is a newly identified forage species known for its remarkable ability to accumulate selenium. While widely distributed across China, the molecular mechanisms underlying selenium metabolism in this species remain poorly understood. *WRKY* transcription factors have been extensively studied for their rolesin regulating plant stress responses. Although genome-wide analyses of *WRKY* genes have been performed in numerous plant species, no such study has been counducted in *B. papyrifera* until now. In this study, 47 *BpWRKY* genes were isolated and screened from the whole genome data of *B. papyrifera* for the first time using bioinformatics analysis. To identify potentially genes involved in selenium accumulation, we integrated physiological indices, qRT-PCR expression data, total selenium content, and miRNA-mediated regulatory networks associated with WRKY genes. Through expression profiling and subsequent subcellular localization experiments, we confirmed the nuclear localization of selected WRKY proteins and identified two putative regulators of selenium metabolism—BpWRKY34 and BpWRKY25.

### Evolutionary characteristics of *BpWRKY* transcription factors and their potential synergistic regulation in response to selenium stress

Research indicates that transcription factors play pivotal roles in plant selenium metabolism. In rice, under As stress, Se affects the expression of *MYB* and *WRKY* transcription factors [21]. In tea plants, the treatment with selenite upregulates the expression of the *MYB* and *bZIP* transcription factors [22]. Recent findings have shown that *HMT* [36], *NAC* [37], *MYB* [38], and *bZIP* [39] transcription factors respond to selenium salt stress in *B. papyrifera*. It is reasonable to assume that the transcription factors *HMT*, *NAC*, *bZIP*, and *WRKY* are synergistically expressed in *B. papyrifera* in response to selenium stress. *WRKY* transcription factors play pivotal roles in responding to biotic and abiotic stresses in plants, primarily by regulating gene expression and participating in diverse signal transduction pathways. Since their initial discovery, *WRKY* genes have become indispensable transcription factor family members [40]. Researchers have found these genes in the genomes of several plant species, including important crops such as *Zea mays* [41] and *Oryza sativa* [42], as well as woody plants such as *Pinus massoniana* [43], *Camellia japonica* [44], and *Salix suchowensis* [45]. Yang et al. identified 174 *WRKY* genes in soybean and 97 in wild rice [46]. This study identified 47 BpWRKY genes from the *B. papyrifera* genome. These genes were classified into three groups based on their WRKY domains [30] and zinc finger structures [31], a categorization consistent with that of the WRKY family in *(A) thaliana* [44]. Overall comparison reveals that the number of WRKY transcription factors in *(B) papyrifera* is relatively low.

The size of gene families is closely associated with evolutionary processes such as gene duplication and genome rearrangement. In the *WRKY* family, gene duplication events have been widely reported across multiple species: Arabidopsis has undergone alpha (α), beta (β), and gamma (γ) three genome duplications [47]., Comparative genomic analysis of homologous D-genome regions between hexaploid wheat Chinese Spring (*Triticum aestivum*) and its diploid progenitor *Aegilops tauschii* revealed substantial sequence divergence exceeding 71% [48], *M. alba* retains 64 *WRKY* genes primarily through paleopolyploidization events [34], among the *WRKY* in *Camellia oleifera* 57 (62.6%) were derived from whole genome duplication (WGD)/fragment duplication [49], and in *Populus euphratica* approximately 83% (86 out of 104) of *WRKY* participated in gene duplication events [50]. The phenomenon of gene duplication in these families is fundamentally rooted in whole-genome duplication events or tight tandem duplications occurring during self-replication on the same chromosome. The latter has been identified as the principal driver of evolutionary processes [42]. Duplicated *WRKY* genes frequently exhibit analogous functions [51], potentially establishing complex and unique regulatory networks [52, 53], and may be coexpressed in response to a singular stressor. Proteins belonging to the same class or subclass typically share similar motifs.

In comparison with the *(A) thaliana* dataset, the *(B) papyrifera WRKY* family demonstrated enhanced conservation, with only a single member (*BpWRKY24*) remaining unclassified, indicating constrained variation and minimal environmental stress during the evolutionary process. The identification of three pairs of highly duplicated genes within the *B. papyrifera WRKY* family suggests their involvement in large-segment genome duplications. The promoters of the *B. papyrifera WRKY* genes were found to contain 105 types of *cis*-elements, including G-box, ABRE, TCA, AuxRR-core, LTR, ARE, and elements associated with biotic or abiotic factors. Notably, *BpWRKY34* and *BpWRKY25*, located in the nucleus, as confirmed by fusion expression vectors, possess an equal number of G-boxes, TCAs, and AREs in their promoters, suggesting that these elements may induce their expression under selenium stress. Interspecies synteny analysis revealed that Arabidopsis has a pair of genes, *AtWRKY4* and *AtWRKY3*, which are homologous to *BpWRKY27*. Previous research has validated the collaborative function of *AtWRKY3* and *AtWRKY4* in *Arabidopsis* [54], where their absence affects the reactive oxygen species (ROS) clearance pathway, consequently diminishing stress resistance [55]. However, based on homology analysis, it is conceivable that the collaborative function of the *BpWRKY* may have diverged or been lost over time.

### The multifaceted roles of selenium in plant physiology and its species-specific regulation of *WRKY* genes

Selenium has a significant impact on plant life, particularly in the context of modern agriculture [56]. Foliar application of nano-selenium enhances photosynthesis, antioxidant activity, and nutrient content in *Salvia miltiorrhiza*, thereby promoting growth [57]. Combined treatment with nano-selenium and melatonin increases glucose and total selenium levels in *Zizyphus jujuba*, with NAC, WRKY, and MYB family genes potentially involved in these enrichment processes [58]; Selenium exposure induces foliar damage in *Allium schoenoprasum* while significantly activating phenylpropanoid biosynthesis and phytohormone signal transduction pathways [59]. In *Brassica oleracea*, selenate treatment leads to a dose-dependent accumulation of selenomethionine (SeMet) and methylselenocysteine (MeSeCys) [60]. Recent studies on *Setaria italica* reveal that Na_2_SeO_4_ accumulation in spikes correlates with upregulated expression of transcription factors such as ABCC13, PHT1.3, SiNRT2.1, glutathione S-transferases (GSTs), and members of the *MYB*, *WRKY*, and *bHLH* families [61].

The presence of Se has been shown to augment a plant’s capacity to withstand abiotic stresses [62, 63], including heavy metals [64]. In *O. sativa*, Se alleviates arsenic-induced phytotoxicity by enhancing antioxidant biosynthesis, reinforcing cell wall integrity, and repairing arsenic-inflicted damage, thereby mitigating growth inhibition and oxidative stress under arsenic exposure [21]. Similarly, Cd exposure, Se exerts catalytic effects to restore chlorophyll content and gas-exchange parameters via elevated antioxidant enzyme activities, effectively reducing Cd-induced oxidative damage in rice [65].

**Fig. 11:**
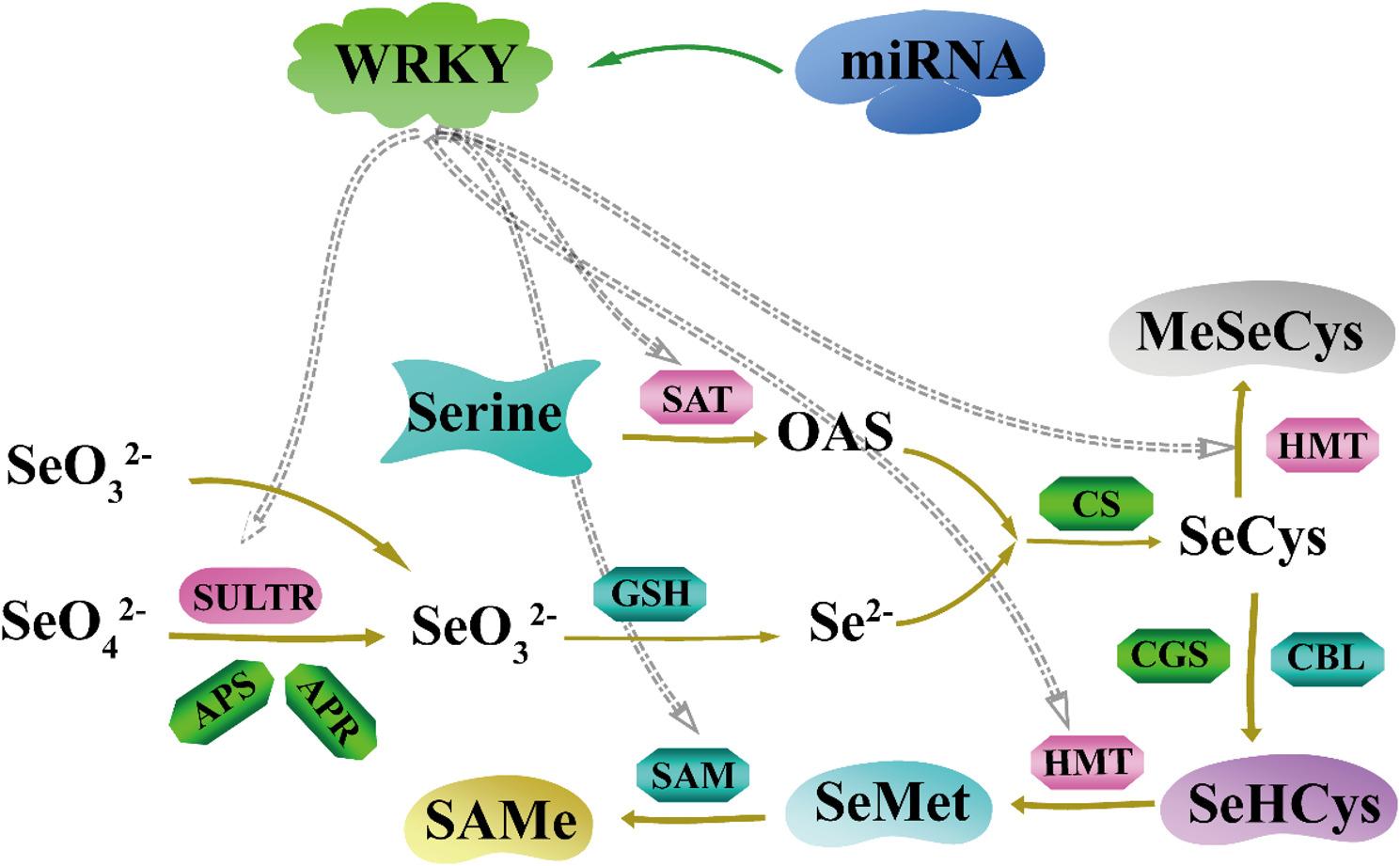
Diagram of selenium metabolism pattern, the dotted line indicates the possible direction of action. O-acetylserine transferase (SAT), Cysteine half-synthase (CS), homocysteine S-methyltransferase (HMT), Cystathionine γ-synthase (CGS), cystathionine β-cleaving enzyme (CBL), S-adenosylmethionine synthase (SAM), Glutathione peroxidase (GSH)

At the biochemical and molecular level, plants predominantly take up inorganic selenium as selenate (SeO₄²⁻) and selenite (SeO₃²⁻). These inorganic selenium species follow distinct metabolic pathways and exert different biological functions, requiring the coordinated action of multiple enzymes (Fig. 11). Previous studies have demonstrated that selenium speciation significantly influences *WRKY* gene expression patterns. For instance, in *C. violifolia*, selenate treatment induced dose-dependent upregulation of *CvWRKY021* and *CvWRKY099*, while selenite treatment caused tissue-specific responses with upregulation in roots but downregulation in shoots [28]. In contrast, the expression pattern of *WRKY* in the *B. papyrifera* is different from this case. The expression levels of *BpWRKY25* and *BpWRKY34* showed progressive upregulation with increasing selenite concentrations, suggesting their specific involvement in selenite-responsive pathways. In comparison, *BpWRKY26* and *BpWRKY45* exhibited elevated expressions with increasing selenate concentration, implicating their distinct roles in selenate uptake or reduction processes. These results not only designate *B. papyrifera* as a reference species for selenium transcription factor studies but also generate essential resources for probing: the molecular basis of selenium species-specific responses, and the hierarchical coordination of selenium metabolic fluxes.

### Candidate *WRKY* genes involved in regulating selenium metabolism in *B. papyrifera*

*WRKY* transcription factors are known to play pivotal roles in regulating plant growth, development, metabolic processes, and stress responses. Phylogenetic analyses have revealed that *WRKY* members within the same subgroup often share conserved functions across species, such as *(A) thaliana*, rice [66], *Triticum aestivum* [67]. This functional conservation enables the prediction of BpWRKY protein roles in *(B) papyrifera* based on characterized homologs in other species. In *A. thaliana*, *AtWRKY33* and *AtWRKY12* function as transcriptional coactivators, activating *RAP2.2* and enhancing hypoxia tolerance [68]. *AtWRKY47*, a key role in maintaining selenium homeostasis and tolerance in *(A) thaliana* [24]. Our phylogenetic analysis (Fig. 1) identified *BpWRKY29* and *BpWRKY37* as close homologs of *AtWRKY47*, clustering within the same evolutionary subclass. This strong phylogenetic relationship suggests these *BpWRKY* proteins may perform analogous functions in selenium metabolism regulation in *(B) papyrifera*. However, potential functional divergence should also be considered given the distinct ecological adaptations of this species.

The *WRKY* family plays a pivotal regulatory role in plant’s salt stress responses [69–71]. Under NaCl treatment, *HcWRKY44* in transgenic *Hibiscus cannabinus* [72] plants exhibited positive induction of genes associated with ABA-regulated signaling, response genes, and stress-related genes. Additionally, *Ginkgo biloba* [57] plants exhibit responsiveness to drought, high temperature, and salt treatments. In *Cunninghamia lanceolata*, *WRKY* is presumed to be involved in the regulatory mechanisms underlying tolerance to low-Pi stress [73]. *WRKY12* and *WRKY13* have been identified in *(A) thaliana* as regulating Cd tolerance positively and negatively, respectively [74, 75]. *WRKY47* has been regarded as a key gene for maintaining selenium homeostasis and tolerance, and selenium stress induces the upregulation of *WRKY47* [24]. These findings also imply that specific *WRKY* genes can enhance salt tolerance [76–78]. In this study, the expression levels of *BpWRKY34* and *BpWRKY25* increased significantly under 400 mg/L selenate stress (Fig. 8), indicating their potential roles in regulating the response of *(B) papyrifera* to selenate.

The miRNAs act as post-transcriptional gene silencers that modulate stress-responsive genes to facilitate plant adaptation to adverse environments [79]. They are known to respond to salt stress across various plant species [80, 81]. For instance, overexpression of AtmiR395c and AtmiR395e in Arabidopsis regulates APS1, APS4, and SULTR2;1, thereby altering sulfate assimilation, transport, and seed germination under salt or drought stress [72]. In Vicia faba, 150 mM NaCl treatment significantly upregulates miRNA287 while downregulating miR243 [82]. Parallel studies in rice revealed selenium-induced upregulation of miR-171, miR-399, and miR-1433, accompanied by downregulation of miR-395 [73]. In this study, bioinformatics analysis identified three key miRNA-WRKY regulatory modules: miR482c-5p::BpWRKY26, miR_237::BpWRKY35, and miR_219::BpWRKY47 (Fig. 8b), with qRT-PCR validating robust negative correlations between these miRNA-mRNA pairs.

Recent studies have clarified the intricate interplay between transcription factors (TFs) and miRNAs in regulating plant responses to selenium stress. Samad et al. [63]. systematically elucidated the cooperative roles of TFs and miRNAs in modulating stress-responsive pathways. In *A. thaliana*, selenium stress specifically activates *WRKY* TFs while suppressing the expression of heavy metal tolerance proteins *HMT1* and *HMT3* [24]. Comparative transcriptomic analyses in Stanleya pinnata revealed selenate-responsive induction of SULTRs, indicating their conserved role in selenium uptake across species [83]. Our correlation analysis demonstrated significantly stronger associations between *PCS* and WRKY candidates compared to SULTR-WRKY relationships (Fig. 9), suggesting preferential functional coupling between *WRKY* and PCS in selenium response. The selenium assimilation pathway involves two critical enzymatic conversions: ATP sulphurylase (APS) catalyzes selenate to 5’-adenosine selenophosphate (APSe) [76], which is reduced to selenite by 5’-phosphorothioate adenosine reductase (APR) [77], the rate-limiting step in selenium assimilation. Subsequent biotransformation into organic selenides is mediated through a coordinated enzymatic cascade involving serine acetyltransferase (SAT) [84], cystathionine γ-synthase (CS), homocysteine methyltransferase (HMT) [36], and S-adenosylmethionine synthase (SAM) [85].

*BpWRKY15*, *BpWRKY18*, *BpWRKY25*,* BpWRKY34*, *BpWRKY37*, and *BpWRKY40*, are highly correlated with the expression of selenium pathway enzymes. We can reasonably infer that *BpWRKYs* participate in Se assimilation processes in *B. papyrifera* by modulating the expression of key metabolic enzymes. These findings collectively demonstrate, that *WRKY* likely function as direct regulators of selenium metabolic genes, while miRNAs fine-tune *WRKY* expression levels through post-transcriptional silencing.

## Limitations and future perspectives

While this study has successfully identified and preliminarily characterized *WRKY* transcription factors with potential involvement in regulating selenium metabolism in *B. papyrifera*, it must be acknowledged that the content of the manuscript has certain limitations. The inferred association between *BpWRKY* genes and the selenium-enrichment phenotype is predicated largely on correlative evidence derived from transcriptomic profiling and qRT-PCR analyses. Such expression-based correlations, while indicative of potential regulatory roles, are insufficient to establish definitive causal relationships. They fail to preclude alternative scenarios—for instance, these *WRKYs* might influence selenium accumulation indirectly through modulation of general stress-response pathways or primary metabolic processes, rather than via direct engagement with selenium-specific metabolic networks. The precise molecular mechanisms by which key regulators such as *BpWRKY34* and *BpWRKY25* modulate selenium metabolism remain unresolved.

Future work will focus on functionally characterizing BpWRKY34 and BpWRKY25 through overexpression and gene editing to decipher their biological roles in selenium accumulation in paper mulberry. Concurrently, we will identify their downstream target genes to elucidate the complete regulatory network. Such efforts will not only clarify the molecular basis underlying selenium hyperaccumulation in *B. papyrifera* but also yield critical genetic resources and theoretical frameworks to advance molecular breeding of selenium enrichment forage crops.

## Conclusion

This study systematically investigated the WRKY transcription factor family in *B. papyrifera* to elucidate its regulatory roles in selenium metabolism. Through genome-wide identification and phylogenetic analysis, 47 *BpWRKY* genes were classified into three major groups. Integrated transcriptomic and physiological analyses identified nine selenium-responsive *BpWRKY* candidates, among which *BpWRKY34* and *BpWRKY25* exhibited the strongest correlations with Se accumulation. Subcellular localization assays confirmed their nuclear enrichment. Notably, co-expression network analysis revealed significant associations between key *BpWRKYs* and selenium metabolic enzymes, while miRNA profiling uncovered post-transcriptional regulation through miRNA and *BpWRKY*. This research will establish a theoretical framework for advancing genetic modification strategies to cultivate selenium-biofortified *B. papyrifera* germplasm through targeted molecular breeding methodologies.

## Materials and methods

### Plant materials and treatments

The one-year-old hybrid *B. papyrifera* ‘Kegou 101’ was planted in the glass greenhouse (30.35 °N, 112.15 °E). The seedlings with the same growth status were randomly divided into seven groups with 3 biological replicates in each group. The leaves were sprayed with 200 µM sodium selenate, 400 µM sodium selenate, 800 µM sodium selenate, 200 µM sodium selenite, 400 µM sodium selenite and 800 µM sodium selenite, respectively. The control group was treated with the same amount of deionized water. The treatment was performed once a week for a total of 4 times. And water is irrigated every three days. The above treatments refer to previously published papers [20, 35, 36], as high selenium concentrations can lead to damaged and blackened, or even wilted leaves of *B. papyrifera*. The leaves of *B. papyrifera* were quickly collected and frozen in liquid nitrogen and stored in a refrigerator at -80 °C for physiological and molecular experiments.

Ninety uniform *B. papyrifera* one-year saplings were randomly allocated into two groups, each consisting of three replicates. In the treatment group, the roots of each sapling were irrigated with 200 mL of a sodium selenate solution (400 mg/L), while the control group received an equivalent volume of deionized water. This irrigation process is repeated three times every 15 days and watered every three days after treatment. After 45 days of treatment, the leaves and roots of each sapling were promptly frozen in liquid nitrogen and stored at − 80 °C for subsequent RNA extraction.

### Identification and analysis of WRKY

This study employed two methods to identify the *WRKY* family in *B. papyrifera*. Initially, the Hidden Markov Model (PF03106) for *WRKY* was acquired from Pfam (http://pfam.xfam.org/). Subsequently, *WRKY* genes were extracted from the *B. papyrifera* genome [36] using HMMER [86]. Target sequences were selected based on a threshold of E-value < 10^− 5^, and nucleotide sequences encoding amino acid residues of less than 35 were excluded. These sequences were subsequently subjected to verification for structural integrity and domain analysis using CDD (http://www.ncbi.nlm.nih.gov/Structure/bwrpsb/bwrpsb.cgi) and SMART (http://smart.embl-heidelberg.de/). Second, plant *WRKY* sequences of the Ref-Seq type were retrieved from the NCBI (https://www.ncbi.nlm.nih.gov). Local BLAST comparisons against the *B. papyrifera* protein database were conducted using TBtools software [87], followed by a structural integrity assessment. Identifying members of the *B. papyrifera WRKY* family was based on the overlap of results obtained from both methods. The basic physicochemical properties of the *WRKY* transcription factors, including molecular weight (MW), isoelectric point (PI), aliphatic index, instability index, and hydrophobicity, were calculated using the Protein Parameter Calc plugins in TBtools. Subcellular localization prediction was performed through the Cell-PLoc 2.0 website (http://www.csbio.sjtu.edu.cn/bioinf/Cell-PLoc-2).

### Phylogenetics, intron-exon structure, motif composition, and cis-acting elements

The *A. thaliana* and *M. alba* WRKY protein sequences were retrieved from TAIR (https://www.arabidopsis.org) and MorusDB (https://morus.biodb.org/morusdb/datasets), respectively. These sequences were aligned using MUSCLE [88], and the resulting alignment was subsequently trimmed using trimAL [89]. A phylogenetic tree was constructed using the maximum likelihood method with IQ-TREE [90], employing a bootstrap value of 1000 iterations. The visualization of the phylogenetic tree was enhanced using the online iTOL (https://itol.embl.de/ ) website.

Sequences spanning 2 kb upstream of the start codon were extracted from the *B. papyrifera* genome for the prediction of promoter *cis*-regulatory elements via PlantCare (http://bioinformatics.psb.ugent.be/webtools/plantcare/html/). The predicted elements were refined using Excel and subsequently visualized with TBtools. Furthermore, gene structures were visualized through the online database Gene Structure Display Server 2.0 (http://gsds.cbi.pku.edu.cn/). Conserved motifs were analyzed in *BpWRKYs* using the online software MEME (http://meme-suite.org/ tools/meme). The motif length was limited to between 6 and 50 amino acids, with a maximum of 10 motifs, and the other parameters were set to their default values.

### Chromosomal collinear analysis

MCScanX software [91, 92] was used to analyze the collinearity of *B. papyrifera WRKY* genome within and between species (*A. thaliana*, *M. alba*), and the collinearity diagram was drawn using the Circos website (http://circos.ca/intro/tabular_visualization/).

### Expression pattern analysis of *BpWRKY* by qRT-PCR

Total RNA was extracted from *B. papyrifera* using a TaKaRa MiniBEST Plant RNA Extraction Kit (Takara, Beijing, China) following the manufacturer’s guidelines. The concentration and quality of the extracted RNA were assessed using a Nanodrop One spectrophotometer (ThermoFisher, Ipswich, USA). Subsequently, reverse transcription into cDNA was performed utilizing PrimeScript™ 1st strand cDNA synthesis kit (Takara, Beijing, China) following the protocol outlined in the manual. Primers were designed based on the coding sequence of the *WRKY* genes (Additional file 5: Table S5), and PCR amplification was conducted with cDNA as the template.

*WRKY* gene expression data under various treatment conditions were extracted from previous transcriptome sequencing results (PowerEdge T640, Dell, China), with a specific focus on the fragments per kilobase per million mapped reads (FPKM) data [20]. Candidate genes potentially involved in selenium metabolism were identified based on a correlation coefficient |>0.5|. For qRT-PCR analysis of these candidate genes, primers were designed using Premier 6.0 (Primer-e Ltd, UK, Additional file 5: Table S5). The Python Ergene package was employed for the screening of ubiquitin as an internal reference gene, following the established methodology outlined in the extant literature [20, 86]. The experimental point samples were processed for three biological replicates and three technical replicates, yielding a total of nine data points. Quantitative real-time polymerase chain reaction (qRT-PCR) was performed using a qPCR Master Mix (purchased from Vazyme, Nanjing, China) according to the provided instructions. Triplicate analyses were performed for each sample on a LineGene 9600 Plus machine (manufactured by Bio, Hangzhou, China). Gene expression levels were then determined using the 2^−ΔΔCT^ method [87].

### Subcellular localization of BpWRKY34 and BpWRKY25

Correlation analysis with selenium content was performed by qRT-PCR, and *BpWRKY34* and *BpWRKY25* with the highest correlation were screened for subcellular localization assay. Prediction analysis of protein sequence revealed that BpWRKY34 and BpWRKY25 were localized in the nucleus. To verify this further, primers were designed (see Table S1) and pNC-Cam1304-35 S::WRKY2-GFP and pNC-Cam1304-35 S::WRKY5-GFP fusion expression vectors with GFP fluorescent proteins were constructed.

The GV3101 strain was resuspended in MS media until the OD_600_ reached 0.5, and 100 µM acetosyringone was added to the infection solution [93]. Precultured *A. cepa* epidermal cells were immersed in an infection solution and shaken at 28 °C for 20 min. Subsequently, the infection solution was removed by placing the *A. cepa* epidermal cells on sterile filter paper. These cells were then transferred to MS coculture medium and incubated in the dark at 25 °C for 24–48 h. Afterward, laser confocal microscopy (TCS SP8 CLSM, Leica, Wetzlar, Germany) was used to observe gene localization. The control experiment employed the use of pCAMBIA1304-35 S::GFP empty vector, while the nuclei that had been stained with 4’,6-diamidino-2-phenylindole (DAPI) dye were utilized as a positive control.

### Detection of total selenium content

The total selenium content was determined by HG-AFS: 0.2 g of selenium powder was weighed, and placed in a tube, and 10 mL of nitric acid and 2 mL of hydrogen peroxide were added. The lid was closed and digestion took place in a microwave for 55 min. After cooling, 5 mL of HCl (6 mol/L) was added to the tube and the solution was heated until it became clear and colorless with the appearance of white smoke, and then cooled. Transfer to a 10 mL volumetric flask and add 2.5 mL of potassium ferricyanide (100 g/L). Add water to make up the final volume. Use Selenium standard solutions of 0, 5, 10, 20 and 30 µg/L to create a standard curve. The selenium lamp requires 60 mA and a 280 V negative high voltage.

### Prediction of the regulatory network of WRKY involved in selenium metabolism

Building on our group’s previous sequencing data [36], we systematically identified 20 key enzymatic genes in the selenium metabolic pathway for quantitative real-time PCR (qRT-PCR) primer design. Constructing co-expression networks between candidate WRKY transcription factors and selenium metabolism-related genes, aims to unravel their regulatory interplay, thereby providing experimental evidence to elucidate the molecular mechanisms through which *BpWRKY* family members participate in plant selenium metabolism.

The TBtools Plant TF Binding Motif Shift program was used to screen and analyze the downstream selenium-related target genes of *BpWRKYs* selenium-related candidate genes and to further validate the relevance of *WRKY* candidate genes to selenium.

### Statistical analysis

The results are presented as the mean ± standard error (SE) from three independent replicates. All data were processed and analyzed using Excel (Microsoft, USA) or Origin Pro 2023 (OriginLab, USA) [89]. Significant differences among multiple groups were assessed by one-way analysis of variance (ANOVA) followed by post-hoc testing using Tukey’s Honest Significant Difference (HSD) test. A *p*-value < 0.05 was considered statistically significant.

## Supplementary Information

Additional file 1.

Additional file 2.

Additional file 3.

Additional file 4.

Additional file 5.

## Data Availability

The datasets generated and analyzed during this study are included in this published article and its supplementary information files. All other relevant data are available from the corresponding author upon reasonable request.

## Abbreviations

Se: selenium
qRT-PCR: real-time quantitative-polymerase chain reaction
MW: molecular weight
PI: isoelectric point
FPKM: fragments per kilobase per million mapped reads
LSD: least significant difference
G-box: light-responsive elements
LTR: low-temperature responsive elements
ARE: anaerobic induction responsive elements
ABRE: elements responsive to abscisic acid
MBS: drought-induced MYB binding sites
